# On the relationship between word reading ability and spelling ability

**DOI:** 10.1007/s11145-024-10566-z

**Published:** 2024-07-02

**Authors:** Rebecca Treiman, Jacqueline Hulslander, Erik G. Willcutt, Bruce F. Pennington, Richard K. Olson

**Affiliations:** 1 Washington University in St. Louis, 1125-0249-02, 1 Brookings Dr, St. Louis, MO 63130 USA; 2 University of Colorado, Boulder, USA; 3 University of Denver, Denver, USA

**Keywords:** Word reading, Spelling, Vocabulary, Individual differences

## Abstract

The goal of the present study was to test theories about the extent to which individual differences in word reading align with those in spelling and the extent to which other cognitive and linguistic skills play different roles in word reading and spelling. Using data from 1,116 children ranging from 8 to 17 years, we modeled word reading and spelling as latent traits with two measures of each skill to reduce measurement error. The models also included five skills that have been theorized to relate differentially to reading and spelling: phonemic awareness, working memory, rapid automatized naming, arithmetic, and vocabulary. The latent-trait correlation for reading and spelling was very high, 0.96, although significantly less than perfect. Vocabulary correlated more strongly with reading (0.64) than spelling (0.56), but the correlations of the other skills with reading and spelling did not differ significantly. Breaking down the sample by age, we found a significantly higher latent-trait correlation between reading and spelling in the younger half (*r* = .98) than in the older half (*r* = .94). This difference may reflect the fact that the words on reading and spelling tests are more different from one another at older ages. Our results suggest that word reading and spelling are one and the same, almost, but that spoken vocabulary knowledge is more closely related to reading than to spelling.

Many theories of literacy and literacy development emphasize the similarities between word reading and spelling (e.g., Ehri, 1997; Kim, 2020; Perfetti, 2017). According to Perfetti’s lexical quality framework, the same representations support both. The higher the quality of a lexical representation, the more accurately and quickly a word can be read and spelled. According to Ehri’s theory of orthographic mapping, the spellings of words are retained in memory as they are read by forming connections between units of the writing system and units of spoken language. These theories suggest that that word reading ability and spelling ability would correlate highly.

At the same time, word reading and spelling differ in some ways. Reading involves accessing or constructing a pronunciation from a letter string, whereas spelling involves accessing or constructing a letter string. The former is easier than the latter, in part because the links from spellings to sounds are more straightforward in English and other writing systems than the links from sounds to spellings (Moll & Landerl, 2009). Moreover, an incomplete or imprecise representation can sometimes allow for accurate reading but is less likely to permit correct performance in a spelling production or recognition task. Such differences lead us to expect that reading and spelling might not correlate all that highly and that certain other cognitive and linguistic skills might correlate differentially with the two. The theory that different lexica are involved in spelling and reading (Weekes & Coltheart, 1996) makes similar predictions.

In the present study, we sought to better understand the relationship between children’s word reading and spelling ability, as measured by performance on standardized tests, and the relationship of reading and spelling to other cognitive and linguistic skills. Previous studies of these issues have used single measures of each skill and are thus limited by the imperfect reliability of the measures. Our participants—1,116 English speakers aged 8 to 17 years—were given multiple measures of word reading, spelling, and other cognitive and linguistic skills. By combining information from several indicators of a skill into a latent variable, we could better estimate the relationships among reading, spelling, and other skills and better test theories about these skills and the links among them.

Our first research question concerned the correlation between spelling accuracy and word reading accuracy. Meta-analyses of studies using single measures of each skill have reported correlations from 0.70 to 0.82 (Ehri, 1997; Kim et al., 2024; Swanson et al., 2003). Our data set included two measures of each of word reading and spelling, allowing us to use latent-trait modeling of their shared variance to deal with measurement error. Would the correlation between reading and spelling now be perfect, as Carver (2003) suggested, or would there be a reasonable amount of independent variance?

Understanding the magnitude of the correlation between word reading and spelling is important not only for theoretical reasons but also because of its implications for children who have difficulty acquiring these skills (Pennington & Peterson, 2015). If word reading and spelling are clearly dissociable even when using latent-trait models, this would support the view that word reading disability and spelling disability should be distinguished and that some children have one disability but not the other (e.g., Fayol et al., 2009; Moll et al., 2014a, b; Moll & Landerl, 2009). A very high or perfect latent-trait correlation between word reading ability and spelling ability would support the view that true dissociations are rare or nonexistent (Joshi & Aaron, 1990).

Our second research question was whether the latent traits for reading and spelling correlate differentially with other cognitive and linguistic skills. If reading ability and spelling ability are one and the same, no such differences would be expected. If somewhat different skills underly reading ability and spelling ability, we would expect certain abilities to correlate more highly with the latent trait for reading and other abilities to correlate more highly with the latent trait for spelling. As we discuss next, the pattern of correlations should shed light on theories about differences between the skills that are involved in spelling and reading.

Enderby et al. (2021) suggested that spelling places higher demands on phonological skills because of the need to segment a word into smaller units and hold them in memory while determining how to spell them. They and other researchers provided some evidence that phonemic awareness is more important for spelling than for reading (e.g., Furnes & Samuelsson, 2011; Landerl & Wimmer, 2008). The meta-analysis of Swanson et al. (2003) also found that phonological awareness correlated more highly with spelling than with reading, although the difference was small (0.45 vs. 0.41) and might have resulted from measurement error. Our study included two measures of phonemic awareness, allowing us to ask whether the latent trait for phonological awareness correlated more highly with the latent trait for spelling than the latent trait for reading.

According to Ehri (1997), another potentially important difference between reading and spelling may be that spelling places more demands on memory. Supporting this idea, the correlation between spelling accuracy and memory span was 0.39 in the Swanson et al. (2003) meta-analysis, higher than the 0.31 correlation between word reading accuracy and memory span that was observed in the same study and the 0.29 correlation between reading and working memory in the later meta-analysis of Peng et al. (2018). Our study included three measures of working memory, allowing us to model working memory as a latent trait and to examine its association with the latent traits for reading and spelling.

Another skill that we investigated was rapid automatized naming (RAN): the ability to quickly access and produce a phonological form given a visual form. Bowers and Wolf (1993) argued that readers who are slow in activating the letters in a word, a slowness that is reflected in slow RAN performance, are less able to encode the letters as a unitary representation. Because more precise orthographic representations are needed for spelling than for reading, RAN may be more closely related to spelling. As discussed by Savage et al. (2008), other theoretical views predict that RAN should relate more closely to reading than to spelling. The meta-analysis by Chen et al. (2021) found that RAN correlated more highly with reading accuracy than with spelling accuracy, but the difference was very small (0.38 vs. 0.35). Our study included four measures of RAN, allowing us to address these issues using latent-trait modeling.

Another skill of interest in the present study was vocabulary. Several theories suggest that knowledge of spoken vocabulary can help readers compensate for partial or incomplete knowledge about spelling-to-sound relations (Steacy et al., 2023; Wegener et al., 2022). For example, a child who does not know the rules involving the use of long and short vowels may nevertheless be able to pronounce the written word “naval” correctly if she knows that the pronunciation with the long first-syllable vowel is an English word and the pronunciation with the short vowel is not. Spoken vocabulary knowledge cannot aid the processes of spelling production or recognition in this way. Consistent with these views, some studies have reported higher correlations between vocabulary and reading than between vocabulary and spelling (Altepeter & Handal, 1985; Braze et al., 2007; Smith et al., 1991; Vance et al., 1985). We had a single measure of vocabulary in our study, and we asked whether it correlated differentially with the latent traits for reading and for spelling.

Because of the increasing focus on co-morbidity between reading and math disabilities (e.g., Pennington & Peterson, 2015), we also examined arithmetic. Zoccolotti et al. (2021) theorized that ability to recall specific instances plays an important role in aspects of mathematical performance and in dealing with words with less predictable spelling–sound mappings. Because predictability is less in the sound-to-spelling direction than the spelling-to-sound direction, we would expect arithmetic to relate more closely to spelling than to reading. Supporting this idea, Moll et al. (2014a, b) reported that children with serious difficulties in arithmetic are likely to have more serious problems with spelling than with reading. We had a single measure of arithmetic in our study, and we asked whether it related more closely to the latent trait for spelling than the latent trait for reading.

Given that the 8- to 17-year age range of our participants is quite large, we also asked whether younger and older children differ in the relationship between word reading and spelling and in the relationships of reading and spelling to other skills. The meta-analysis of Kim et al. (2024) showed a significantly higher correlation between word reading and spelling for children in prekindergarten to Grade 2 than for adults, although no significant difference between children in prekindergarten to Grade 2 and older children. The weaker relationship between word reading and spelling in adults, Kim et al. suggested, reflects the fact that many adults have reached asymptotes in reading and spelling and so there is less intra-individual variability.

To summarize, our first goal was to examine the relationship between word reading and spelling using latent-trait modeling, an approach that has not previously been used in this area. Our second research question was about how the latent traits for word reading and spelling relate to other cognitive and language skills. Our third question was whether the relationship between word reading and spelling and the relationship between each of these and other cognitive skills is different for younger and older children.

## Method

### Participants

The 1,116 participants were part of an ongoing study at the Colorado Learning Disabilities Research Center (CLDRC) that recruits twin pairs from across the Colorado Front Range using records from 27 different school districts and the Colorado Twin Registry. All pairs of twins with a broadly defined school history of reading difficulty and/or attention deficit hyperactivity disorder (ADHD) for at least one member of each pair are invited to participate, as are a subset of pairs with no school history of either type of problem. Twin pairs are excluded if either member is learning to read English as a second language, has documented brain injury, seizures, sex chromosome anomalies, or a rare genetic condition such as Williams Syndrome, or has uncorrected hearing or visual impairment.

The inclusion of twin pairs in the CLRDC sample has supported behavior-genetic analyses of the genetic and environmental etiology of reading, spelling, and related skills (e.g., Friend et al., 2007). For example, Olson et al. (2018) conducted behavior genetic analyses of latent traits for reading and spelling in a sample of monozygotic and dizygotic twins that partly overlapped with the twins in the present study. Individual differences in reading (h^2^ = 0.74) and spelling (h^2^ = 0.71) were highly heritable, and their genetic correlation was also high (*r*_a_ = 0.97).

In the present study, we focus on the phenotypic correlations between reading, spelling, and related skills by randomly including just one twin from each of 1,116 twin pairs aged between 8 and 17 years (*M* = 11.25, *SD* = 2.4) for whom we have complete data on the measures examined here. The sample included 579 males and 537 females, and they were tested between 1993 and 2015. The participants were drawn from 379 monozygotic pairs and 737 dizygotic pairs. The random selection of one participant from each pair yielded 240 children with a school history of reading difficulty only, 132 children with a school history of ADHD only, 114 children with a school history of both disorders, and 586 children with no school history of reading difficulty or ADHD. The remaining 44 participants were tested before the twin pairs were assessed for ADHD. Ten of these children had a school history of reading difficulty and the other 34 did not. Almost all the children, 98.8%, were reported by their parents to be Caucasian. Other racial identities included African American (2.8%), Asian (2.2%), Hawaiian/Pacific Islander (0.2%), and other (3.3%). For 1.4% of the children, no information about race was available. (These numbers sum to more than 100% because information about race and ethnicity was reported by both parents and because, for children tested after 2006, parents could select more than one identity). The percentage of children who were reported to be Hispanic was 10.4%. The study was approved by the Institutional Review Board at the University of Colorado, Boulder. Informed consent was obtained from children’s parents or guardians and assent from the children themselves.

### Procedure

Participants completed two 2.5-hour testing sessions on one day and two on another. Care was taken to reduce stress and fatigue during the test sessions, including by the provision of breaks. The measures for the present study were a subset of the full test battery and were distributed across the four testing sessions.

### Measures

Our measures include some that have been standardized in normative populations with reliabilities reported by the tests’ publishers. In these cases, we report the published reliabilities. For these tests, and for experimental measures that have not been standardized, we also report low-bound reliability estimates based on the tests’ correlations among monozygotic twin pairs, who share both their genes and family environment. We calculated these reliability estimates using data from the members of the monozygotic pairs who were included in the present study together with data from the other member of each pair. This method allows for a comparison of low-bound reliability estimates across standardized and non-standardized measures.

### Word reading

#### Reading recognition

We used the reading recognition subtest of the Peabody Individual Achievement Test-Revised (PIAT-R), which includes 66 words of increasing difficulty that are printed on a card (Markwardt, 1989, 1998). The participant is asked to read the words aloud in sequence until they miss five of the last seven words or reach the end of the list. We used the standard score in our analyses (reliability as reported by test publisher = 0.96; *r*_MZ_ = 0.82).

#### Timed oral reading of single words

This test (Olson et al., 1994) assesses word recognition accuracy for a series of 182 increasingly difficult single words presented on a computer screen. For a response to be scored as correct, it had to be initiated within 2 s. The dependent variable in our analyses was the number of words correct (*r*_MZ_ = 0.86). There is an algorithm to score past the final word when the ceiling criteria have not been met.

### Spelling

#### Spelling production

We used the spelling subtest of the Wide Range Achievement Test-Revised (Jastak & Wilkinson, 1984), which requires participants to write the correct spellings of words. Level 1 of the test, for children under the age of 12, includes 45 words. Level 2, for children older than 12, includes 46 words. Each word was spoken individually by the examiner and used in a sentence. The words are presented in increasing order of difficulty, and testing stops after a child makes 10 consecutive errors. We used the standard score in our analyses (alternate form reliability as reported by publisher = 0.90; *r*_MZ_ = 0.88).

#### Spelling recognition

We used the spelling recognition test of the PIAT-R (Markwardt, 1989, 1998). In this test, words are presented orally by the examiner, both individually and in sentences. The participant is asked to select the correctly spelled word from among four choices that are shown on a card. These choices are likely to be pronounced like the target word when read aloud, meaning that correct performance requires knowledge of the specific orthographic form of the target. There are 70 target words that are presented in increasing order of difficulty, the first of which is a practice item. The task is discontinued if the participant makes five errors in any seven consecutive responses. We used the standard score in our analyses (alternate form reliability as reported by publisher = 0.88; *r*_MZ_ = 0.72).

### Working memory

#### Digit span

We used the digit span test from the Wechsler Intelligence Scale for Children-Revised (WISC-R, Wechsler, 1974) or the Wechsler Intelligence Scale for Children-Third Edition (WISC-III, Wechsler, 1991). This test requires participants to repeat increasingly long strings of digits spoken by the examiner. We used the scaled score in our analyses (reliability as reported by publisher = 0.78 for WISC-R and 0.85 for WISC-III; *r*_MZ_ = 0.58 for WISC-R and 0.49 for WISC-III).

#### Sentence span

In this test (Siegel & Ryan, 1989), participants provide the last word for a set of sentences read by the examiner (e.g., “I throw the ball up and then it comes…”) and then must reproduce the words that they provided after all sentences in that set have been completed. The task begins with three trials with a set size of two. The difficulty is then increased by adding one sentence at each subsequent level until the individual fails all trials at a particular set size. Our dependent variable was the number of sentence-ending words recalled correctly (*r*_MZ_ = 0.42).

#### Counting span

In this task (Case et al., 1982), the participant counts aloud the number of yellow dots on a set of cards. After all cards in a set are completed, the participant is asked to recall, in order, the number of yellow dots that appeared on each card in the set. The task begins with three trials with a set size of two. The difficulty is then increased by adding one card at each subsequent level until the individual fails all trials at a particular set size. The dependent variable was the number of sets recalled correctly (*r*_MZ_ = 0.45).

### Rapid automatized naming

These tests are adaptations of those used by Denckla and Rudel (1974, 1976). For each test, the participant is instructed to scan from left to right an array of items that are shown on a card and to say the items’ names as rapidly as possible. Fifteen seconds are allowed for each test, and the dependent variable is the total number correct. The present version of the task has been shown to account for significantly more variance in several reading measures than the version used by Denckla and Rudel (Compton et al., 2002).

#### RAN pictures

This test uses line drawings of familiar objects. There are 10 rows of pictures, with five pictures per row (*r*_MZ_ = 0.44).

#### RAN colors

This test contains 10 rows of colored circles with 10 circles in each row. Red, blue, green and yellow colors are randomly distributed within each row (*r*_MZ_ = 0.52).

#### RAN numbers

This test uses 15 rows of randomly distributed numbers (1, 2, 4, 6, 7, and 9), each row with five items. Participants are instructed to name as many numbers as possible (*r*_MZ_ = 0.55).

#### RAN letters

This test contains 15 rows of randomly distributed letters (a, b, d, o, p, and s), each row with five items (*r*_MZ_ = 0.55).

### Phonemic awareness

#### Phoneme segmentation and transposition

In this complex phonemic awareness task, as described in Gayán and Olson (2001), participants are asked to remove the first sound of a word spoken by the examiner, put it at the end of the word, and then add the sound /e/. There are nine practice and 45 test items. The dependent variable is a weighted percentage correct measure. Participants get most credit for a perfectly correct response and less credit if the first phoneme is correctly moved but the response contains other errors or if the first letter or the entire onset is moved, in cases where this yields a different response from moving the first phoneme (*r*_MZ_ = 0.68).

#### Phoneme deletion

In this task, Part 1 of the phoneme deletion task described in Gayán and Olson (2001), participants hear a nonword which they are asked to repeat. Participants are then asked to remove a specified phoneme from the nonword and are told that, if this is done correctly, the result is a word. There are a total of 40 trials, and the score is the percent of correct responses (*r*_MZ_ = 0.78).

### Arithmetic

We used the arithmetic test from the WISC-R (Wechsler, 1989) for children tested between 1993 and 2005 and the WISC-III (Wechsler, 1991) for children tested between 2006 and 2015. These are timed tests that include orally administered arithmetic word problems. We used the scaled score in our analyses (reliability as reported by publisher = 0.77 for WISC-R and 0.78 for WISC-III, *r*_MZ_ = 0.61 for WISC-R and 0.63 for WISC-III).

### Vocabulary

This was the 32-item vocabulary test from the WISC-R (Wechsler, 1989) for children tested between 1993 and 2005 and the 30-item vocabulary test from the WISC-III (Wechsler, 1991) for children tested between 2006 and 2015. These tests ask participants to define words presented orally by the examiner. Children receive 2 points for a response that demonstrates knowledge of the meaning (e.g., “something you cut with” for “knife”), 1 point for an answer that shows partial knowledge (e.g., “eat with it” for “knife”) and 0 points for a wrong or very vague answer (e.g., “I have one” for “knife”). Testing is discontinued if a child receives 0 points on five consecutive items on the WISC-R and four consecutive items on the WISC-III. We used the scaled score in our analyses (reliability as reported by publisher = 0.86 for WISC-R and 0.87 for WISC-III; *r*_MZ_ = 0.77 for WISC-R and 0.84 for WISC-III).

## Results

We begin by presenting descriptive statistics and correlations. We then report a factor analysis to check that the working memory measures load on the same factor and that the same is true for the RAN measures and the phonemic awareness measures. The central questions of the study are then addressed through latent-trait models.

### Descriptive statistics

Table 1 presents the mean, standard deviation, and range for each measure, as well as the maximum possible scores for the nonstandardized tests. Note that the means and standard deviations for the standardized reading and spelling tests—the reading recognition test, the spelling production test, and the spelling recognition test—are very close to the means (100) and standard deviations (15) for the norming samples.

**Table 1:** Descriptive statistics for standardized and nonstandardized tests and maximum scores for nonstandardized tests

Measure	Mean (SD)	Range	Maximum
Reading recognition	103.99 (12.74)	65–135	
Timed oral reading of single words	114.93 (46.14)	11–192	192
Spelling production	99.97 (16.79)	58–151	
Spelling recognition	101.95 (13.41)	65–135	
Digit span	9.96 (2.95)	1–19	
Sentence span	5.22 (2.14)	0–15	15
Counting span	6.68 (2.47)	0–15	15
RAN picture	19.51 (3.99)	6–34	50
RAN color	22.82 (5.72)	2–48	100
RAN number	32.91 (7.66)	3–63	75
RAN letter	30.79 (8.09)	3–58	75
Phoneme segmentation and transposition	74.66 (22.28)	0–100	100
Phoneme deletion	68.55 (23.07)	0–100	100
Arithmetic	10.86 (3.28)	1–19	
Vocabulary	11.47 (3.11)	1–19	

### Correlations

Prior to performing the correlational analysis and the subsequent analyses, the phoneme segmentation and transposition variable was square root transformed to reduce its skew. This ensured that skewness and kurtosis were less than **+/–**1 for all variables. All variables were then regressed on the linear, quadratic, and cubic effects of age, standardized within sex, and trimmed at +/–3 standard deviations. Table 2 shows the correlations among the resulting measures. As the table shows, the individual measures for word reading and spelling were all highly correlated. The spelling production measure was more highly correlated than the spelling recognition measure with the two word reading measures, possibly due to the higher reliability of the spelling production measure.

**Table 2:** Correlations for individual measures

Variable	1	2	3	4	5	6	7	8	9	10	11	12	13	14	15
1. Reading recognition	1.00														
2. Timed oral reading of single words	0.87	1.00													
3. Spelling production	0.84	0.86	1.00												
4. Spelling recognition	0.74	0.75	0.79	1.00											
5. Digit span	0.50	0.48	0.49	0.42	1.00										
6. Sentence span	0.41	0.39	0.36	0.33	0.42	1.00									
7. Counting span	0.38	0.35	0.37	0.33	0.47	0.45	1.00								
8. RAN picture	0.25	0.25	0.20	0.22	0.15	0.22	0.20	1.00							
9. RAN color	0.37	0.34	0.33	0.29	0.30	0.26	0.32	0.48	1.00						
10. RAN number	0.34	0.37	0.38	0.33	0.25	0.17	0.27	0.40	0.56	1.00					
11. RAN letter	0.47	0.50	0.49	0.44	0.29	0.23	0.29	0.43	0.53	0.70	1.00				
12. Phoneme segmentation and transposition	0.64	0.60	0.63	0.52	0.46	0.34	0.35	0.12	0.30	0.25	0.32	1.00			
13. Phoneme deletion	0.73	0.72	0.74	0.57	0.49	0.39	0.38	0.15	0.32	0.32	0.40	0.75	1.00		
14. Arithmetic	0.53	0.54	0.54	0.50	0.47	0.38	0.40	0.23	0.33	0.30	0.35	0.43	0.49	1.00	
15. Vocabulary	0.60	0.61	0.53	0.51	0.38	0.41	0.30	0.23	0.24	0.17	0.28	0.41	0.44	0.50	1.00

Note. All correlations significant at *p* < .001, one tailed

### Factor analysis

To examine the factor structure of the variables, we fit a factor analysis using SPSS, using maximum likelihood extraction and oblimin rotation to allow for correlated factors. Only the first three factors had Eigenvalues greater than 1, but inspection of the scree plot supported the inclusion of a fourth factor. As Table 3 shows, the first factor in the pattern matrix was defined largely by the four reading and spelling measures. The second factor was defined by the four RAN measures. The third factor was defined by the two phonemic awareness measures, and the fourth factor was defined by the three working memory measures. The arithmetic and vocabulary measures cross-loaded on both the literacy and working memory factors. The four extracted factors explained 61.8% of the variance before rotation. Table 4 shows the factor correlation matrix.

**Table 3:** Pattern matrix from factor analysis

Measure	Factor 1	Factor 2	Factor 3	Factor 4
Reading recognition	0.77	0.04	0.17	0.04
Timed oral reading of single words	0.85	0.07	0.12	–0.04
Spelling production	0.77	0.08	0.22	–0.07
Spelling recognition	0.81	0.07	0.01	–0.05
Digit span	0.14	0.04	0.20	0.46
Sentence span	0.09	0.00	0.04	0.60
Counting span	–0.04	0.14	0.12	0.56
RAN picture	0.05	0.51	–0.16	0.15
RAN color	–0.07	0.63	0.06	0.19
RAN number	–0.03	0.87	0.09	–0.11
RAN letter	0.20	0.77	0.04	–0.13
Phoneme segmentation and transposition	0.15	0.00	0.62	0.19
Phoneme deletion	0.18	0.04	0.73	0.13
Arithmetic	0.32	0.10	0.06	0.34
Vocabulary	0.60	–0.07	–0.11	0.29

**Table 4:** Factor correlation matrix

Factor	1	2	3	4
1	1.00			
2	0.43	1.00		
3	0.60	0.27	1.00	
4	0.52	0.37	0.32	1.00

### Latent-trait models

The loading of both the spelling and reading measures on the first factor indicates their close relation, as do the correlations for the individual spelling and reading measures in Table 2. However, even those high correlations may have been attenuated by measurement error. This problem can be mitigated through latent-trait modeling of the shared variance for the two spelling measures and the shared variance for the two reading measures. The inclusion of spelling and reading as separate factors in the latent-trait model allowed us to examine their relationship with one another and their relationship with other factors. The model also included latent traits for working memory, RAN, and phonemic awareness, in line with the results of the factor analysis and the correlational analyses. Because we had a single measure for each of vocabulary and arithmetic, the model had seven factors: reading, spelling, working memory, RAN, phonemic awareness, vocabulary, and arithmetic.

**Fig. 1:**
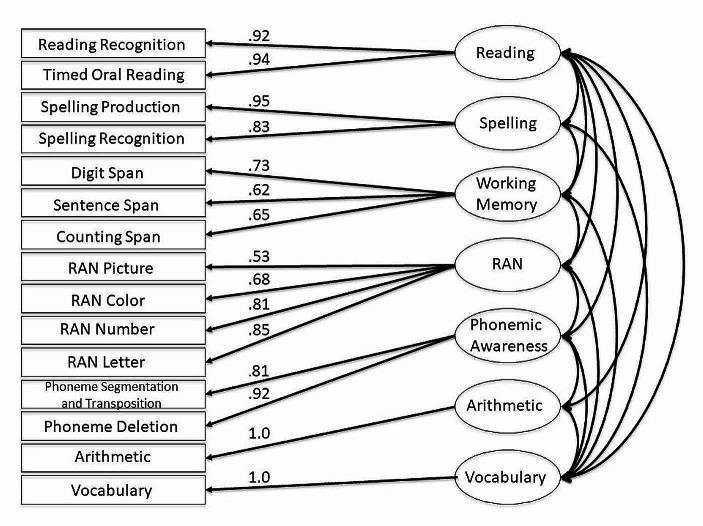
Standardized factor loadings for confirmatory factor analysis

Figure 1 shows the results of the model as fit using the OpenMx package in R (Version 2.20.6, Neale et al., 2016). The overall fit statistic for models using raw data in OpenMx, log likelihood, is sensitive to the scale of the data and is not, on its own, interpretable. However, nested models can be compared by calculating the change in the measure of model deviance − 2LL, which approximates a chi-square distribution. Fit of the base model can be assessed via the comparative fit index (CFI) and the root-mean-square error of approximation (RMSEA). CFI compares the current model with a null model. In line with Hu and Bentler (1999), models with CFI values between 0.95 and 1.00 are considered good fits to the original data. RMSEA is a population-based index that estimates the discrepancy in fit between the model and data, taking into account degrees of freedom. Loehlin (1998) suggests that RMSEA values less than 0.10 show that a model has good fit. The model fit statistics for the seven-factor model with five latent traits and two individual measures indicated that the model provided a satisfactory fit to the data across the full age range (*N* = 1116, 16,678 *df*, −2LL = 37007.42, CFI = 0.97, RMSEA = 0.06).

Table 5 shows the correlations for the latent traits in the seven-factor model, along with those for the single measures of vocabulary and arithmetic. A key result is the latent trait correlation between reading and spelling of 0.96. Although the correlation between these two latent traits is very close to 1, it could not be set to 1 without significant loss of fit (Δ−2LL = 38.90, Δ1 *df*, *p* < .001).

**Table 5:** Correlations among latent variables and single variables

	Reading	Spelling	Working memory	RAN	Phonemic awareness	Arithmetic
Spelling	0.96					
Working memory	0.67	0.65				
RAN	0.55	0.55	0.49			
Phonemic awareness	0.83	0.82	0.70	0.46		
Arithmetic	0.58	0.58	0.63	0.41	0.53	
Vocabulary	0.64	0.56	0.53	0.30	0.48	0.50

Note. All correlations significant at *p* < .001, one tailed

The correlations of reading and spelling with phonemic awareness (0.83, Δ − 2LL = 2.34), working memory (0.66, Δ − 2LL = 2.40), RAN (0.55, −2LL < 1), and arithmetic (0.58, Δ − 2LL < 1) could be equated without significant loss of fit (all *p* > .05, Δ*df* = 1), indicating that these variables correlated equally with reading and spelling. The single measure of vocabulary was the only factor in the model that had a significant difference in its correlations with the latent trait for reading (*r* = .64) and the latent trait for spelling (*r* = .56). Equating the vocabulary correlations with reading and spelling resulted in a significant loss of fit (0.61, Δ − 2LL = 48.68, Δ*df* = 1, *p* < .001). Thus, vocabulary is more highly correlated with reading than with spelling, although both correlations are significant and substantial.

To address our question about whether the relationship between word reading and spelling and the relationship between each of these and other cognitive skills is different for younger and older children we ran separate models for participants below and above the median age of 10.6 years. The age means for the two groups were 9.2 years and 13.2 years, respectively. The two groups of participants were similar in gender and racial breakdown and the percentage with a history of ADHD, although the younger group had more children with a history of reading disability (37% vs. 29%). Table 6 shows the factor correlations for the two groups. The correlation between the latent variables for reading and spelling was significantly higher for the younger group (*r* = .98) than the older group (*r* = .94; *p* < .001 for the difference), and both correlations were significantly less than 1 (young: Δ − 2LL = 8.97, Δ*df* = 1, *p* < .01; old: Δ − 2LL = 33.97, Δ*df* = 1, *p* < .001). There were no significant differences for the reading and spelling correlations with working memory, RAN, phonemic awareness, or arithmetic for either age group (all: Δ − 2LL < 3.65). In both groups, vocabulary correlated significantly more highly with the latent variable for reading than with the latent variable for spelling (young: Δ − 2LL = 12.83, Δ*df* = 1, *p* < .001; old: Δ − 2LL = 35.47, Δ*df* = 1, *p* < .001).

**Table 6:** Correlations among latent variables and single variables for younger and older groups

	Reading		Spelling		Working memory		RAN		Phonemic awareness		Arithmetic	
	Young	Old	Young	Old	Young	Old	Young	Old	Young	Old	Young	Old
Spelling	0.98	0.94										
Working memory	0.65	0.69	0.65	0.66								
RAN	0.64	0.48	0.63	0.48	0.51	0.47						
Phonemic awareness	0.84	0.84	0.82	0.81	0.70	0.70	0.50	0.44				
Arithmetic	0.58	0.58	0.59	0.57	0.62	0.64	0.43	0.40	0.55	0.51		
Vocabulary	0.58	0.72	0.52	0.62	0.50	0.56	0.32	0.27	0.43	0.56	0.48	0.52

Note. All correlations significant at *p* < .001

The comparison of correlations for the older and younger groups was supported by the fact that both group’s scores were age adjusted and normally distributed, thus meeting a fundamental assumption of our latent trait models. However, a reviewer argued that we should also compare correlations between groups of high and low readers based on their reading standard scores. This is problematic, partly because the oppositely skewed within-group distributions for reading and related skills would not meet the normal distribution assumption of our latent trait models. Nevertheless, in a supplemental analysis, we ran separate models for participants below and above the standard score of 100 on the PIAT word recognition test. The high (*r* = .93) and low (*r* = .92) reader latent trait correlations between reading and spelling were not significantly different, and they were slightly lower than the correlations for the normally distributed high and low age groups (*r* = .94 and *r* = .98 respectively).

## Discussion

The present study addressed three questions about individual differences in word reading and spelling ability: how closely they relate to one another, whether they relate differentially with other cognitive and linguistic skills, and whether there are differences in these relationships for younger and older children. In what follows, we discuss our evidence on these questions and its implications for theory and practice.

### How closely associated are word reading accuracy and spelling accuracy?

The present study is the first, to our knowledge, to use latent-trait modeling to examine the strength of the relationship between spelling and word reading. The correlations between the individual measures of reading and spelling in our sample of twins range from 0.75 to 0.87 (Table 2), a similar range as in past studies using samples of primarily non-twins (Ehri, 1997; Kim et al., 2024; Swanson et al., 2003). The correlation between the latent variables is much higher: 0.96 when considering the data from our full sample. An even more extreme example of the impact of use of latent variables comes from studies of the association between reading comprehension and listening comprehension. The single-measure correlations between tests of reading comprehension and listening comprehension averaged about *r* = .50 in Quinn and Wagner’s (2018) meta-analysis. The correlation was much higher, 0.94, in a latent-trait model of the common variance across multiple measures of each construct (Christopher et al., 2016).

The very high latent-trait correlation between reading and spelling speaks against the theory that reading and spelling involve different lexica (Weekes & Coltheart, 1996) and in favor of the view that the same representations underlie both (Ehri, 1997; Perfetti, 2017). According to these latter theories, orthographic representations are retained in memory as words are read. These representations, which include connections between units of spelling and units of language, underlie both reading and spelling. However, contrary to Carver’s (2003) view, the correlation between reading and spelling is not perfect. This result indicates that word reading and spelling involve some different skills—a different enough mix that children’s ranking relative to one another in terms of word reading does not perfectly match their ranking in spelling.

The very high latent-trait correlation between reading and spelling that we observed is important practically as well as theoretically. When we informally asked researchers and educators of our acquaintance to estimate the correlation between perfectly reliable and valid tests of word reading and spelling for 8- to 17-year-old learners of English, the mean estimate was 0.70, well below the value of 0.96 that we obtained in the present study. If educators and clinicians underestimate the association between reading and spelling, they may be too quick to consider children as having deficits only in reading or only in spelling. Such children may not receive the right mix of instruction.

### Do reading and spelling correlate differently with other cognitive and linguistic skills?

To determine which variables related to word reading and spelling might be responsible for the very modest independent variance that we observed between the two skills, we examined five variables for which there are theoretical reasons to expect different correlations with reading and spelling. Four of the variables’ correlations with reading and spelling were not significantly (*p* < .05) different despite our high statistical power to detect a difference.

One variable for which we found no significant difference in correlations with reading and spelling was phonological awareness. The meta-analysis of Swanson et al. (2003) showed a small tendency for phonological awareness to correlate more highly with spelling than with reading, consistent with the idea that phonological skills play a larger role in spelling than in reading (Enderby et al., 2021). However, we did not find such a difference.

A second variable that showed no significant difference in correlations with word reading and spelling was the working memory latent trait. We included this variable to test the idea that that spelling places greater demands on memory than reading (Ehri, 1997). Although Swanson et al.’s (2003) meta-analysis showed a slightly higher correlation between spelling accuracy and memory span than between reading and memory span, we did not find such a difference.

Some views of RAN (Bowers & Wolf, 1993) suggest that it should correlate more highly with spelling accuracy than with word reading accuracy, while other views discussed by Savage et al. (2008) predict the opposite. Our latent trait for RAN did not correlate significantly differently with the latent traits for word reading accuracy and spelling accuracy, speaking against theories about RAN that predict a difference.

Moll et al. (2014a, b) reported that children with serious difficulty in arithmetic tended to have greater difficulty with spelling than with reading, a result that may reflect the importance of memory for specific instances in arithmetic and in spelling (Zoccolotti et al., 2021). However, our measure of arithmetic did not correlate differently with word reading and spelling. We acknowledge that an arithmetic measure that placed more stress on recall of specific instances might show such a difference.

Of the five skills that we considered, the only one to correlate differentially with our reading and spelling latent traits was vocabulary knowledge. Vocabulary was significantly more correlated with reading than with spelling: *r* = .64 as compared to 0.56 based on data from the full sample. This difference was also found in the separate analyses of the younger and older children. Some investigators (Altepeter & Handal, 1985; Braze et al., 2007; Smith et al., 1991; Vance et al., 1985) have noted higher correlations of vocabulary with word reading accuracy than with spelling accuracy but have not discussed possible reasons for the difference. There are several reasons, not mutually exclusive, why vocabulary may correlate more highly with word reading than with spelling. One reason is that readers can sometimes use their knowledge of spoken vocabulary to avoid mispronouncing difficult words (Wegener et al., 2022). Spoken vocabulary knowledge cannot aid the spelling process in the same way. A second reason for the higher correlation of vocabulary with reading than with spelling may be that children can sometimes predict at least part of a word’s spelling when they learn the word orally, before they have seen it in writing. These expectations, called orthographic skeleta, may help them read the words when they encounter them in print (Wegener et al., 2018). Although a partially correct orthographic skeleton may help a child read a word, it is less likely to support correct spelling. A third reason that oral vocabulary correlates more highly with word reading ability than with spelling ability may be that exposure to uncommon words in texts helps children learn the meanings of the words but is not always sufficient to permit correct spelling. These factors may play a larger role for older children than for younger children, as evidenced by the finding (see Table 6) that the correlations of vocabulary with reading ability and with spelling ability were larger in older children than in younger ones.

### Differences between younger and older children

We found that the correlation between word reading and spelling is even larger for 8- to 10 ½-year-olds (*r* = .98) than for 10 ½- to 17-year-olds (*r* = .94). The only previous meta-analysis to have examined possible differences in the reading–spelling correlation as a function of age found a significantly higher correlation between reading and spelling for children in prekindergarten to Grade 2 than for adults, but no significant difference between children in prekindergarten to Grade 2 and older children (Kim et al., 2024). Kim et al. suggested that the lower correlation between reading and spelling in adults may reflect the fact that many adults are close to ceiling in reading and spelling and so there is little intra-individual variability. In our study, however, there was substantial variability in performance in the older group. As we discuss in more detail below, the correlation between reading and spelling may be lower in older children because of differences in the characteristics of words on reading tests and spelling tests. Because children are generally better at reading words than at spelling them, reading tests must include harder words than spelling tests to avoid ceiling and floor effects, if scoring is based on accuracy. The differences between the types of words on reading tests and spelling tests may be larger at older ages.

To test these ideas, we identified the last word that a child would need to get correct on each reading and spelling test to obtain the mean score observed in our sample for the each of the six-month bins centered at 9.2 years (the mean age for the younger group), 10.6 (the mean age for the whole sample), and 13.2 years (the mean age for the older group). In identifying the last word correct, we assumed that a child would perform correctly on all the words up to this word and perform incorrectly on the words after it. We considered this a reasonable simplification given the words were designed to increase in difficulty across the tests. We identified a band of 15 words that included the last correct word and the 7 words before and after it. Our assumption was that the words in this band would be ones that distinguish better performers from poor performers at the age of interest. As one measure of word difficulty, we calculated the average U value (frequency per million words weighted for dispersion of words across content areas) for the words in each band from Zeno et al. (1995), which includes words in reading materials targeted at U.S. students in the primary grades through college. Table 7 shows the mean frequency of the words in each band for each age and type of test. The table also shows the mean number of letters in the words in each band.

**Table 7:** Mean frequency and length of words in bands for different ages on reading and spelling tests

Test type	Frequency			Length		
	Age 9.2	Age 10.6	Age 13.2	Age 9.2	Age 10.6	Age 13.2
Spelling	111.1	57.7	36.9	6.6	7.7	8.9
Reading	18.6	4.7	1.2	7.1	7.9	8.4

The results in Table 7 show, not surprisingly, that the words in the critical bands are less common and longer at the higher age levels than the lower age levels for both reading and spelling. Most important for present purposes, the words on the spelling tests are much more common than the words on the reading tests and the difference in frequency is particularly large at older ages. Specifically, the spelling words are 6 times more common than the reading words at 9.2 years, l2 times more common at 10.6 years, and 32 times more common at 13.2 years. We found the same pattern of results when we considered just the WRAT spelling test and the PIAT reading recognition test, the two standardized tests in which examinees produce a spelling or a pronunciation without time pressure. We suspect that standardized tests other than those used here would show the same patterns. This, we suggest, may help explain the significantly higher correlation between reading and spelling for younger children.

### Implications for children with disabilities

Difficulties with the basic skills of word reading and spelling contribute to difficulties with reading comprehension and writing, and questions have arisen about how to classify children who have trouble acquiring these basic skills (Pennington & Peterson, 2015). Some have argued that educators and clinicians should distinguish between word reading disability and spelling disability and that some children have one disability but not the other (e.g.Fayol et al., 2009; Moll et al., 2014a, b; Moll & Landerl, 2009). However, the very high latent-trait correlation between word reading and spelling observed here suggests that true dissociations are rare (Joshi & Aaron, 1990). Word reading problems and spelling problems usually go hand in hand, and children with problems in both reading and spelling need instruction and practice in both.

### Implications for other languages

The present study was conducted in English, which has a complex writing system. Both reading ability and spelling ability are typically assessed in English in terms of accuracy, facilitating comparison between the tasks. Such comparisons are more difficult for languages with simpler links between letters and phonemes, such as Finnish and Italian. In these languages, reading performance is typically assessed using timed measures, to avoid ceiling effects, whereas spelling is typically assessed using accuracy. The difference in method of assessment is likely to lower the correlation between reading and spelling and affect their relationships with other skills (e.g., Moll et al., 2014).

### Limitations of the present study

The present study is characterized not only by its use of English but also by its use of twins who were over-sampled for a school history of reading difficulty and/or ADHD in at least one member of each pair. The fact that our sample that was normally distributed with means and variances on standardized tests that are close to the publishers’ standardizing population norms suggests that our results would generalize to non-twin samples who were not over-sampled for reading difficulty and/or ADHD. However, future research is needed with these populations.

The minimal number of manifest variables for each latent trait is two, and three or more is preferable (Streiner, 2006). We had three manifest variables only for our rapid naming and memory latent traits. Our latent traits for reading, spelling, and phonemic awareness were based on two measures each, although this is not a major limitation in the present study since the manifest variables for each of these latent traits were highly correlated. The single measures of vocabulary and arithmetic could not be modeled as latent traits, so measurement error likely constrained their correlations with reading and spelling. Future studies should ideally include at least three measures for all latent traits.

## Conclusion

Our results support the idea that word reading and spelling are “one and the same, almost” (Ehri, 1997). Word reading and spelling correlate almost perfectly with one another when using latent-trait modeling to deal with measurement error, and their correlations with several other cognitive and linguistic skills are statistically indistinguishable. Oral vocabulary is the one skill that we found to correlate differentially with reading and spelling. The results support the idea that word reading and spelling rely on the same representations (Ehri, 1997; Perfetti, 2017) but that vocabulary knowledge can sometimes compensate for lower-quality representations to support reading (Wegener et al., 2022).
